# Genetic and Virulent Difference Between Pigmented and Non-pigmented *Staphylococcus aureus*

**DOI:** 10.3389/fmicb.2018.00598

**Published:** 2018-04-03

**Authors:** Jing Zhang, Yujuan Suo, Daofeng Zhang, Fangning Jin, Hang Zhao, Chunlei Shi

**Affiliations:** ^1^MOST-USDA Joint Research Center for Food Safety, School of Agriculture and Biology, Shanghai Jiao Tong University, Shanghai, China; ^2^Institute for Agri-Food Standards and Testing Technology, Shanghai Academy of Agricultural Science, Shanghai, China

**Keywords:** *Staphylococcus aureus*, staphyloxanthin, non-pigmented, virulence, murine sepsis model

## Abstract

Staphyloxanthin (STX), a golden carotenoid pigment produced by *Staphylococcus aureus*, is suggested to act as an important virulence factor due to its antioxidant properties. Restraining biosynthesis of STX was considered as an indicator of virulence decline in pigmented *S. aureus* isolates. However, it is not clear whether natural non-pigmented *S. aureus* isolates have less virulence than pigmented ones. In this study, it is aimed to compare the pigmented and non-pigmented *S. aureus* isolates to clarify the genetic and virulent differences between the two groups. Here, 132 *S. aureus* isolates were divided into two phenotype groups depending on the absorbance (OD_450_) of the extracted carotenoids. Then, all isolates were subjected to *spa* typing and multilocus sequence typing (MLST), and then the detection of presence of 30 virulence factors and the gene integrity of *crtN* and *crtM*. Furthermore, 24 typical *S. aureus* isolates and 4 *S. argenteus* strains were selected for the murine infection assay of *in vivo* virulence, in which the histological observation and enumeration of CFUs were carried out. These isolates were distributed in 26 sequence types (STs) and 49 *spa* types. The pigmented isolates were scattered in 25 STs, while the non-pigmented isolates were more centralized, which mainly belonged to ST20 (59%) and ST25 (13%). Among the 54 non-pigmented isolates, about 20% carried intact *crtN* and *crtM* genes. The *in vivo* assay suggested that comparing with pigmented *S. aureus*, non-pigmented *S. aureus* and *S. argenteus* strains did not show a reduced virulence in murine sepsis models. Therefore, it suggested that there were no significant genetic and virulent differences between pigmented and non-pigmented *S. aureus*.

## Introduction

*Staphylococcus aureus* is an aggressive pathogen, which is responsible for a wide array of hospital- and community-acquired infections, ranging from pyogenic skin infections to complicated life-threatening diseases, such as bacteremia and endocarditis (Liu et al., 2008; Singh, 2014). More than 90% of *S. aureus* isolates produce the pigment staphyloxanthin (STX), thus make them present pigmented (Pelz et al., 2005; Mishra et al., 2011).

*Staphylococcus aureus*, especially the antibiotic-resistant strains, is closely related to high morbidity and mortality in nosocomial infections caused by septic shock and severe sepsis. Compared to methicillin-susceptible *S. aureus* (MSSA), methicillin-resistant *S. aureus* (MRSA) infections will lead to higher mortality, morbidity and economic loss, which have brought a heavy burden on healthcare around the world (Kong et al., 2016). Over the past several decades, there has been an evolution of multidrug-resistant bacteria due to the long-term usage of antibiotics, causing current usage of bactericidal compounds often unsuccessful (Cegelski et al., 2008; Lee et al., 2014). The limitation of antibiotics necessitates the search for novel therapeutic approaches that do not have the potential to induce drug resistance. In recent years, anti-virulence therapies have received great attention, which are regarded as an alternative approach that disturb bacterial toxins or virulence factors and/or their pathway of production (Kong et al., 2016). Rather than cellular viability and bacterial growth, anti-virulence approaches targeting bacterial virulence may be less possible to develop drug resistance (Lee et al., 2012, 2014).

Anti-virulence approaches are divided into two types, a direct approach, targeting *S. aureus* toxins and an indirect approach, targeting pathways that are involved in toxin production (Kong et al., 2016). In previous studies, there were many compounds being identified as effective anti-virulence candidates, for example, peptide variants of autoinducing peptides (AIPs) have been proved to inhibit the virulence of MRSA. What’s more, based on the thiolactone structure, the cyclic AIP mimetics have been synthesized to restrain the accessory gene regulator histidine kinase (AgrC) function *in vitro* (George et al., 2008); A family of small-molecule compounds, phenolic ingredients in traditional Chinese medicinal herbs such as β-cyclodextrin, have been discovered to inhibit α-hemolysin (Hla) *in vitro* production, thus decrease *S. aureus* colonization, in addition, some of them could also remarkably repress the transcription of toxin genes *sea, seb* and *tsst-1* (Khodaverdian et al., 2010; Qiu et al., 2010a,b; Shoham, 2011).

Since the STX is regarded as an important virulence factor, among those anti-virulence approaches, the STX biosynthetic pathway is one of the druggable targets against infections. There are many studies focused on the inhibition of STX production for the therapy of *S. aureus* infection. As an orange-red triterpenoid, membrane-bound carotenoid, STX is important in the environmental fitness of *S. aureus* (Clauditz et al., 2006). STX can improve the antioxidant properties of *S. aureus* and the resistance to neutrophils. The early enzymatic steps in STX production are similar to those of cholesterol biosynthesis. The first step in STX biosynthesis is catalyzed by the dehydrosqualene synthase CrtM, resulting in the formation of dehydrosqualene (4, 4′-diapophytoene) by the head-to-head condensation of two molecules of farnesyl diphosphate. The dehydrosqualene desaturase CrtN dehydrogenates dehydrosqualene to form the yellow, main intermediate 4, 4′-diaponeurosporene, which is then further oxidized, glycosylated, and esterified to give the carotenoid, STX (Pelz et al., 2005; Liu et al., 2008). Liu et al. (2008) found that the phosphonosulfonates (and related bisphosphonates), a cholesterol biosynthesis inhibitor, can inhibit CrtM by binding to it, resulting in the increased susceptibility to neutrophil killing and to innate immune clearance of non-pigmented bacteria in a mouse infection model. It was found that naftifine (Chen et al., 2016) and NP 16 (Gao et al., 2017) can both block the biosynthesis of carotenoid pigment by competitively inhibiting *S. aureus* CrtN and then attenuate the virulence of these *S. aureus* isolates as well as increase their susceptibility to innate immune clearance.

While, it is worth emphasizing that there are still 10% of *S. aureus* isolates from human infections are non-pigmented. It might be attributed that in these isolates the STX biosynthetic relevant genes are missing or fail to express correctly. Because STX confers resistance against oxidative stress and neutrophil killing (Liu et al., 2005), and the inhibition of STX synthesis has been demonstrated to attenuate the virulence of *S. aureus* (Liu et al., 2008), we hypothesize that the non-pigmented *S. aureus* strains would have attenuated virulence compared to pigmented ones because of the lack of STX production.

Therefore, the current study aimed to investigate differences in virulence and genotype between the pigmented and non-pigmented *S. aureus* strains. With this aim, multilocus sequence typing (MLST) and *spa* typing have been used to characterize the genotype of the *S. aureus* strains which have been divided into two groups depending on the absorbance (OD _450_) of extracted carotenoids. Then, virulence factors profiles, molecular typing features and *in vivo* infection grades were compared based on the two groups.

## Materials and Methods

### Staphylococcal Strain

A total of 132 *S. aureus* isolates were obtained from various samples in Shanghai and Ningbo of China. Among these isolates, 74 have clinic relevance, originated from samples such as swab anal, hands of doctors and food samples that caused food poisoning; the others were obtained from food-processing plants and food samples, such as milk, meats, and vegetables, randomly purchased from local grocery stores. Isolation and identification of *S. aureus* were performed as previously described (Wang et al., 2012). Four *S. argenteus* strains were added in murine infection assay, including SJTUF21164, isolated from hands of healthy person by Shanghai CDC; SJTUF20124 and 20419, isolated from food poisoning samples by Ningbo CDC, Zhejiang Province; SJTUF28299 (DSM28299), a type strain isolated from blood. Genomic DNA of each isolate was purified using TIANamp Bacteria DNA Kit (TIANGEN Biotech, China) following the manufacturer’s instructions, with the addition of lysostaphin (Sigma-Aldrich, United States) for bacterial lysis. DNA amount and purity were tested with an Nanodrop-2000 Spectrophotometer (Thermo Fisher Scientific, United States) (Song et al., 2016).

### Quantification of Pigment and Grouping

Staphyloxanthin and intermediate carotenoids were extracted from all the *S. aureus* isolates. Cells were grown at 37°C for 24 h in 0.5 L of tryptic soy broth, TSB (Oxoid Ltd., Basingstoke, United Kingdom). Cells were harvested by centrifugation (5,000 *g*, 10 min) and washed twice with phosphate-buffered saline, PBS (Thermo Fisher, China). Cultures were centrifuged, and the cell pellets were either used immediately or stored at -20°C (Marshall and Wilmoth, 1981).

Extraction and quantification of carotenoids were carried out as previously described (Liu et al., 2005). Stationary-phase (24 h) cultures of S. aureus were equilibrated for growth yield and then subjected to methanol extraction (Marshall and Wilmoth, 1981). Five grams (wet weight) of washed cells was resuspended in 15 mL of methanol, heated in a water bath at 55°C for at least 5 min while being stirred gently, until all the visible pigment of bacteria were extracted and dissolved into methanol. Then, the methanol extract liquid was cooled and centrifuged. Pigment content was then quantified by the absorbance profile of carotenoids as determined spectrophotometrically at an optical density of 450 nm (OD_450_) (Mishra et al., 2011). The S. aureus isolates were divided into two groups (pigmented and non-pigmented) depending on the OD_450_ results.

### Multilocus Sequence Typing (MLST)

All the *S. aureus* isolates were further characterized by MLST. Seven *S. aureus* housekeeping genes (*arcC, aroE, glpF, gmk, pta, tpi*, and *yqiL*) were amplified by PCR, and DNA sequencing was performed for all of the PCR products. DNA sequences were assembled as well as the length proofreading and homology alignment with reference sequences in SeqMan (Lasergene 8, DNASTAR, Madison, WI, United States) and analyzed on the MLST website^1^ (Song et al., 2016). The phylogenetic tree based on MLST types were constructed with MEGA v.5.2.1.

### *spa* Typing

The *spa* typing was performed as mentioned above (Harmsen et al., 2003). Briefly, following the PCR amplification of the repeat-containing region of the staphylococcal protein A gene (*spa*), DNA sequencing of the PCR products was carried out. The *spa* repeats and types were determined using the Ridom Spa Server^2^. If a *spa* repeat did not match any *spa* types in the database, the sequence of the *spa* was identified as a new type (Song et al., 2015).

### Detection of Virulence Factors

The presence of 30 virulence factors in *S. aureus* isolates were determined by simplex PCR amplification, using previously described primers (Supplementary Table S1), which are present in non-core genome (Thammavongsa et al., 2015). These virulence factors included staphylococcal enterotoxin (SE) genes (*sea∼see, seg∼seu*, and *sey*), exfoliative toxin genes (*eta, etb*, and *etd*), Panton–Valentine leukotoxin gene (*PVL*), toxic shock syndrome toxin genes (*tsst-1*), chemotaxis inhibitory protein of *Staphylococcus* genes (*chp*), collagen binding protein genes (*cna*), staphylokinase genes (*sak*) and von Willebrand factor-binding protein genes (*vWbp*).

### Analysis of crtM and crtN

The non-pigmented *S. aureus* isolates were further tested by simplex PCR amplification of *crtM* and *crtN* genes (Supplementary Table S1) as previously described (Zhang et al., 2016). All of the PCR products were analyzed by DNA sequencing and the mutation sites were identified by gene sequence alignment.

### Murine Infection Assay

This study was carried out in accordance with the recommendations of the Guide for the Care and Use of Laboratory Animals of the Shanghai Jiao Tong University. The protocol was approved by the Committee on the Ethics of Animal Experiments of the Shanghai Jiao Tong University (Permit Number: A2016103). BALB/c-NU male mice of SPF level, 5–6 weeks old and 20–25 g weighted (Slac, Shanghai, China) were selected for this assay. Mice were injected i.p. with 10^8^ CFU/g of exponential phase bacteria in 200 μL PBS as previously described (Deshmukh et al., 2009) and observed every 12 h to record their symptoms and behaviors. Six mice were used for each *S. aureus* isolate. All mice were killed at day 3, and moribund mice were killed ahead of time (Tong et al., 2013).

Kidneys, spleens, and livers were harvested. Half of these organs were homogenized for enumeration of live bacterial cells as colony forming unit (CFU) as previously described (Deshmukh et al., 2009). The average CFU of each group (6 mice) was recorded after the over variant samples had been excluded. The rest of these organs were fixed in 10% buffered formalin and frozen at -80°C for histology observation.

All the above experiments were performed in our BSL-2 lab and complied with the corresponding operation rules.

### Statistical Analysis

SPSS v.18.0 (SPSS) was used for statistical analysis. Differences in the distribution of virulence genes in the different phenotypes were analyzed by Pearson’s *Chi-square* test (two-tailed). The difference is regarded significant if the *P*-value is less than 0.05. MEGA v.5.2.1 was used for UPGMA dendrogram construction.

## Results

### Diversity of Pigmented and Non-pigmented Isolates

**FIGURE 1:**
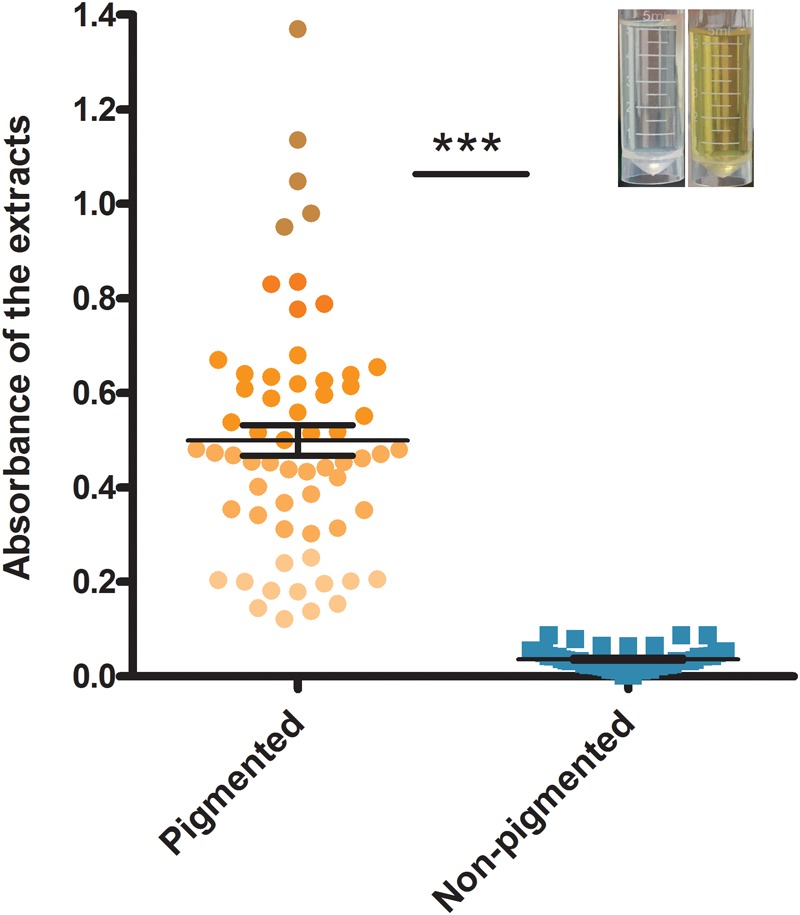
The grouping result according to the absorbance (OD_450_) of carotenoids extracted from *S. aureus* strains. Bars represent mean CFUs and standard error of measurement (SEM), and ^∗∗∗^ indicates there is a significant difference (*P* < 0.05) compared with the other group. Blue points represent non-pigmented strains, and yellow points represent pigmented strains; the deeper points the higher absorbance is.

The isolate grouping according to the carotenoid content is shown in **Figure 1**. Absorbance (OD_450_) of the carotenoids ranged from 0.027 to 1.136. During the early visual observation of these isolates’ colony phenotypes, all the apparently white colonies, which later were determined with a mutated *crtMN* gene, had OD_450_ less than 0.100, however, the slightly yellow colonies had OD_450_ more than 0.100. Thus, 0.100 was set as the OD_450_ threshold for non-pigmented isolates. Those isolates with OD_450_ less than 0.100 were grouped into non-pigmented group (41%, 54/132) and the rest fell into pigmented (59%, 78/132). The carotenoids content of isolates from two groups are significantly different (*P* < 0.05). Among the 54 non-pigmented strains, 41 (76%) have clinic relevance.

**FIGURE 2:**
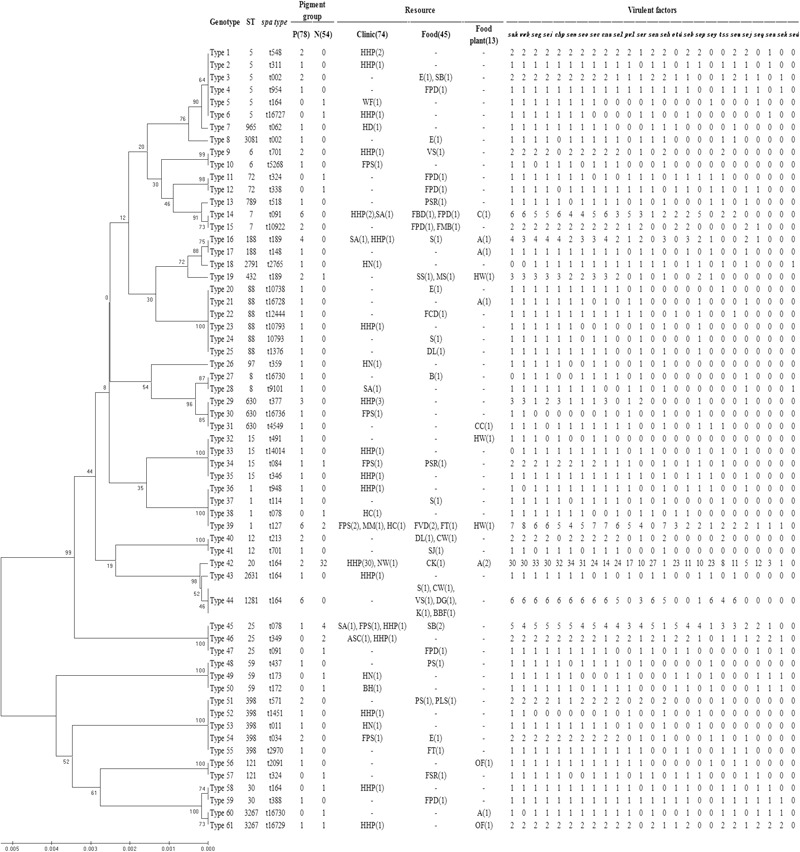
Dendrogram of MLST types and genotypic relationship of 132 examined strains. UPGMA dendrogram displays clustering and genetic similarity (using Jaccard’s coefficient) of MLST genotype profiles. For each genotype, MLST, *spa* types, pigment group, resource and virulence factors are listed. The number either in or outside parentheses represents the number of *S. aureus* isolates. In the column of “Pigment group,” P represents pigmented and N represents non-pigmented. In the column of “Resource,” A, apparatus; ASC, anal swabs of cook; B, bullfrog; BBF, boneless beef brisket; BH, barrow in the hospital; C, chairs; CC, clothes cuff; CK, cooked chicken; CW, chicken wing; DG, duck gizzard; DL, duck leg; E, eggs; FBD, frozen beef dumpling; FCD, frozen chive dumpling; FMB, frozen meat bun; FPD, frozen pork dumpling; FPS, food poisoning sample; FSR, frozen spring roll; FT, frozen Tangyuan; FVD, frozen vegetable dumpling; HC, hands of cooker; HD, hands of doctor; HHP, hands of healthy person; HN, hands of nurse; HW, hands of worker; K, kelp; MM, MRSA monitor; MS, melon seeds; NW, nursing workers; OF, operating floor; PLS, pork leg with skin; PS, pork with skin; PSR, pork side ribs; S, sirloin; SA, swab anal; SB, steamed bread; SJ, salted jellyfish; SS, sea shell; VS, vegetable salad; WF, water faucet.

The dendrogram of MLST types was shown in **Figure 2** along with the result of *spa* typing, pigment groups, resource and virulence factors. The isolates that showed the same MLST and *spa* type were grouped into one genotype, thus, the 132 isolates fell into 61 genotypes in the dendrogram, representing 26 sequence types (STs) and 49 *spa* types. According to the results, some ST types confer a single *spa* type, such as ST20 (t164), ST1281 (t164) and ST432 (t189). It also happened that one ST has multiple *spa* types, such as ST5 (t002, t164, t311, t548, t16727), ST88 (t1376, t10738, t12444, t16728) and ST398 (t011, t034, t571, t1451, t2970). On the contrary, some *spa* types confer a single ST, such as t127 (ST11) and t078 (ST25); some *spa* types have multiple STs, such as t164 (ST5, ST20, ST30, ST1281, ST2631), t002 (ST5, ST3081), t091 (ST7, ST25) and t701 (ST6, ST12).

Then, the correlation of genotype with pigment of *S. aureus* isolates was analyzed. The non-pigmented group had less variety of MLST types comparing to the pigmented group. The pigmented isolates had 25 ST types, while the non-pigmented isolates only had 11 ST types, among which, ST20 was the dominant one (59%, 32/54) following by ST25 (13%, 7/54), as shown in **Figure 2**. Among the 61 genotypes, the pigmented group scattered in 49 types, while the non-pigmented in only 18 types. And the Type 42 (ST20, t164) was dominant (26%, 34/132) following by Type 39 (ST1, t127) (6%, 8/132), and Type 14 (ST7, t091)/Type 44 (ST1281, t164) (both were 5%, 6/132).

### Prevalence of Virulence Factors

**FIGURE 3:**
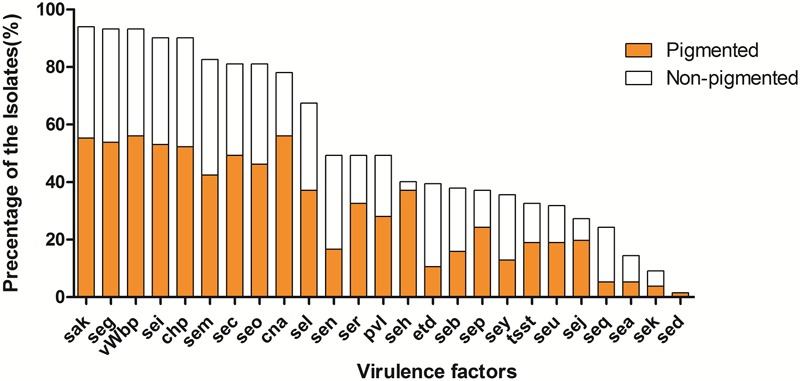
The prevalence of virulence factors in two phenotypic groups of strains. The percentage of all strains for each gene was calculated based on the pigment of each strain. Pearson’s chi-square test (two-tailed) was used to test the difference in the distribution of virulence genes among strains belonging to different pigment groups.

The prevalence of virulence factors in two pigment groups is shown both in **Figures 2, 3**. Each isolate carried at least one gene of virulence factor, and 3 isolates carried 22 genes at the most. About 40% (53/132) of examined isolates, representing 35% (27/78) pigmented and 48% (26/54) non-pigmented carried more than 15 genes of virulence factors. Isolates that carried 12 or 15 virulence factors accounted for the first and second largest proportion, with 13% (17/132) and 12% (16/132), respectively.

The *vWbp*-encoding gene was the most prevalent (93%) in all the examined isolates; representing 56% pigmented isolates and 37% non-pigmented, without significant difference between the two groups. PVL-encoding gene (*PVL*) and TSST-encoding gene (*tsst-1*) were found in 65 (49%) and 43 (33%) isolates, respectively. The SE genes, *seg, sei, sem, sec*, and *seo* were found in most of the isolates (81–93%); the adhesion-associated genes, *sak* and *cna* were also highly prevalent (94% and 78%, respectively). Some of the 30 virulence factors were rare [*sed* (2%), *sek* (9%), *sea* (14%)] in the examined isolates. In addition, 5 (*see, ses, set, eta* and *etb*) of them were either absent, thus they didn’t appear in **Figure 3**. Most of the virulence factors showed no difference between the two pigment groups, except *seh* gene (3% for non-pigmented versus 37% for pigmented) and *seq* (19% for non-pigment versus 5% for pigment). It seems that STX production has no significant correlation with the virulence factors.

### *In Vivo* Virulence in Murine

We selected 24 isolates (12 were pigmented and 12 were non-pigmented) of *S. aureus* from the 132 examined isolates, which are similar with each other in ST types, *spa* types and virulence factors in pair. In addition, we also selected 4 *S. argenteus* isolates to make a comparison.

After infection, those mice lost weight significantly during the following 3 days, mostly decreasing from 20–25 g to 15–17 g. A series of distinct symptoms appeared, such as shortness of breath, slow action, and dull hair. Some of them were even with eye abscess and bloody stool. The severity of these apparent symptoms was consistent with subsequent pathological examination.

**FIGURE 4:**
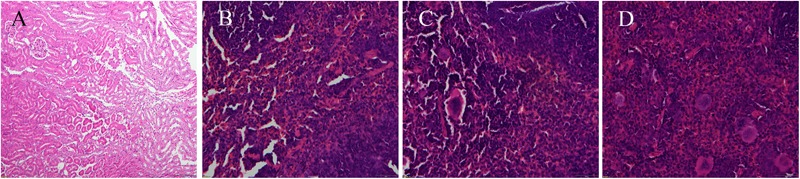
Photograms of histological sections of representative splenic lesions infected by different *S. aureus* strains. **(A–D)** Shows the histological sections of representative lesions for different infection severity levels from weak, medium, strong to very strong, respectively.

**FIGURE 5:**
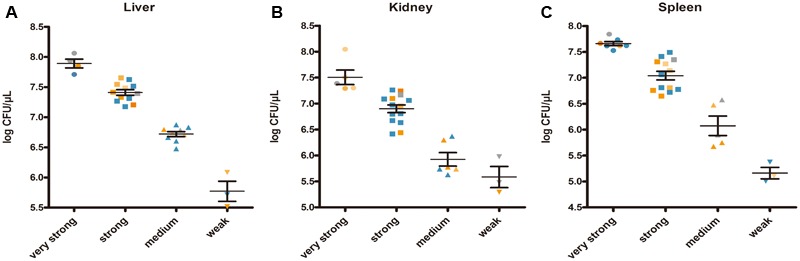
*Staphylococcus aureus* CFUs recovered from abscess lesions (**A** for liver, **B** for kidney, and **C** for spleen). **(A–C)** Shows the CFUs of different infection severities. Bars represent mean CFUs and SEM. Gray points represent for *S. argenteus*; blue points represent for non-pigmented strains, and yellow points represent for pigmented strains; the deeper points the more pigment the strains have.

Enumeration of live bacterial cells as CFUs was conducted as soon as the mice were sacrificed. Liver, spleen and kidney tissues were collected from each mouse. The live bacterial quantification recovered from various tissues proved that each mouse was invaded and infected with different amount of *S. aureus*. Depending on the severity of the degeneration and edema (**Figure 4**), the infection was divided into 4 grades (weak, medium, strong, and very strong), and the corresponding CFUs of each grade were shown in **Figure 5**. The infection grades of 28 examined strains are shown in **Table 1**. For each strain, STX level, *crtMN* integrity and *in vivo* infection grades are listed. Both pigmented and non-pigmented isolates caused the four grades of infection, and their live bacterial amounts were positively correlated with the infection grades.

**Table 1:** The infection grades of 28 examined strains.

ID^a^	Genotype	STX levels^b^	Intact *crtMN*	Infection grades^c^		
			(Y/N)	Liver	Kidney	Spleen
AU-1	Type 39	3	Y	2	1	1
AU-2	Type 38	0	Y	1	2	2
AU-3	Type 39	0	N	1	2	3
AU-4	Type 39	3	Y	3	1	2
AU-5	Type 39	0	Y	3	2	3
AU-6	Type 39	2	Y	2	2	2
AU-7	Type 19	3	Y	1	0	1
AU-8	Type 19	0	N	2	2	2
AU-9	Type 5	0	Y	2	2	2
AU-10	Type 1	3	Y	0	2	2
AU-11	Type 6	0	Y	2	1	0
AU-12	Type 3	2	Y	0	1	1
AU-13	Type 34	0	Y	1	2	3
AU-14	Type 34	5	Y	2	2	3
AU-15	Type 42	1	Y	1	3	2
AU-16	Type 42	0	N	0	1	3
AU-17	Type 42	1	Y	1	3	2
AU-18	Type 42	0	N	1	2	2
AU-19	Type 45	2	Y	2	3	3
AU-20	Type 45	0	N	2	1	0
AU-21	Type 61	0	N	1	2	2
AU-22	Type 61	1	Y	2	3	0
AU-23	Type 48	3	Y	2	2	2
AU-24	Type 49	0	N	2	2	2
AR-1	Type 62	0	N	3	2	2
AR-2	Type 63	0	N	1	0	1
AR-3	Type 64	0	N	3	3	3
AR-4	Type 65	0	N	2	0	1

*^*a*^AU represent *S. aureus*, AR represent *S. argenteus*. ^*b*^The number 0–5 represent STX level, the larger the number, the more STX there is. Zero means no STX detected. ^*c*^The infection grade 0–3 mean weak, medium, strong and very strong, respectively.*

Some isolates, such as SJTUF 21027 (*S. aureus*) (belonging to Type 39) and SJTUF 21164 (*S. argenteus*) (belonging to Type 64), carried less than 4 kinds of virulence factors, but still caused a strong infection. It seems that no matter how many virulence factors those isolates carried but at least one of the virulence factors including *vWbp, PVL* and *tsst-1*, they could cause strong infections. Neither virulence factors nor infection grades had obvious relationship with ST and *spa* types, as well as the pigment groups.

## Discussion

### The Influence of Deficiency of Pigment Production

According to the current results, among the 132 examined *S. aureus*, 41% (54/132) of them did not produce STX, making them present non-pigmented during natural *in vitro* culture. It has been reported that over 90% *S. aureus* isolates produce STX before, while in the current study, a similar number of pigmented and non-pigmented isolates were selected deliberately for better comparison on their virulence. It is notable that the extension of cultivation time, bacterial subculture as well as the addition of oxidant (such as FeCl_3_) to TSB did not induce them to product STX (data not shown). What’s more, the 12 non-pigmented isolates that were used for *in vivo* assay did not change their final phenotype on the plate after infection, which means they show a stable non-pigmented phenotype whether *in vitro* or *in vivo*. As it reported that the STX isn’t a fatal deficiency for *S. aureus* since the most virulence traits are not essential for bacterial survival (Rasko and Sperandio, 2010), which make means that the lack of STX did not influence their survival in natural *in vitro* culture as well as *in vivo* infections. The analysis about the source information of isolates showed most of the non-pigmented strains has clinic relevance, indicating a different source between the two phenotypic groups.

Golden pigment STX is regarded as an important feature of the human pathogen *S. aureus* that increase the bacterial resistance to an innate host immune against infection, the oxidation-based clearance. In response to changing host environments, *S. aureus* has the capability to switch on selective sets of genes to enhance its chances for survival (Cheung et al., 2004). Given STX’s antioxidant properties, it will possibly increase the survival of microbes and offer evolutionary adaptation to various environments. Despite some studies that demonstrated the positive effect of STX on bacterial survival due to the fact that STX stabilized ROS (Liu and Nizet, 2009), it is not a matter of growth, which is proved by Lan et al. (2010), who observed the omission of a growth limiting amino acid did not enhance pigmentation of *S. aureus*. It makes sense that both pigmented and non-pigmented *S. aureus* can survive in nature and cause diseases.

### The Genetic Diversity

Traditionally, MLST and *spa* typing have been used to characterize the genotype of *S. aureus*. In this study, although the distribution of non-pigmented isolates is more concentrated, found in 42% (11/26) ST types, almost every ST type (25/26) contain pigmented isolates even in the ST types which non-pigmented isolates held the dominant position, suggesting the genetic diversity of the two phenotypic groups are of no dramatic difference.

Many studies revealed that the knockout of *crtM* or *crtN* could significantly repress the production of STX (Liu et al., 2008; Liu and Nizet, 2009). Thus, the block of the STX biosynthesis process has proposed as an alternative anti-virulence therapy, in which *crtM* and *crtN* are target for drugs used in the treatment of infections caused by pigmented *S. aureus* (Wang et al., 2016).

Nevertheless, in our study, among the 54 non-pigmented isolates, there are about 20% of them carrying complete *crtN* and *crtM* genes without any sequence mutation or deletion, still demonstrated non-pigmented phenotypes. These results are corresponded with previous reports that not all the non-pigmented isolates are lack of integrated *crtN* or *crtM*. Since the production of them are regulated by the metabolic pathways including purine biosynthesis, the TCA cycle, and even oxidative phosphorylation (Marshall and Wilmoth, 1981). These associated genes did exist but would usually not normally express.

### The Diversity of the Virulence Factors

*Staphylococcus aureus* can produce various virulence factors, related to severe pneumonia, soft tissue infections and staphylococcal scalded skin syndrome. Among those virulence factors, heat-stable enterotoxin (SE), is undoubtedly one of the primary causes of staphylococcal food poisoning (Becker et al., 2003). Before the *sey* was added in 2015, there were 21 identified SEs and staphylococcal-like enterotoxin (SEl) genes, namely *sea* to *see, seg* to *sev* (Xie et al., 2011).

Because lots of virulence genes are coordinately regulated in *S. aureus*, it is reasonable to assume that genes acting on the production of pigmentation may also affect the express of virulence factors and consequently influence the bacterial pathogenicity (Lan et al., 2010).

The detection of virulence factors can not only prevent the genetic diversity, but also indicate the different pathogenicity of *S. aureus* isolates. In this study, these examined *S. aureus* isolates carried various virulence factors, which further suggested their different genotypes. In comparison, pigmented isolates were the dominant isolates that carried the *seh* gene. While in general, there was no significant difference of virulence factors between the two phenotype groups, which demonstrated that their pigment phenotype had no correlation with the presence of virulence factors as well as their pathogenicity.

The pathogenesis of staphylococcal infections is a multifactorial process. It is on account of the production of various virulence factors, which are controlled by multiple regulatory systems and affected by environmental and nutritional signals (Somerville and Proctor, 2009). In order to adapt to the incessant environmental changes for their own survival and causing infection, bacterium can adjust the high degree of variability in the production of toxic genes by a complex network modulated by factors such as the *agr* locus (RNAIII), SarA, and SigB (Clauditz et al., 2006; Lee et al., 2012).

### The Diversity of the *in Vivo* Virulence of Two Pigment Groups

*Staphylococcus argenteus* is a novel *Staphylococcus* species, which is closely associated with *S. aureus* (Zhang et al., 2016). This species presents a non-pigmented phenotype on chocolate agar plates due to the lack of the gene cluster responsible for STX production (Argudin et al., 2016). For comparison, 4 *S. argenteus* strains were added to the *in vivo* virulence assay. The choice of the 24 *S. aureus* isolates involved in the *in vivo* assay was non-biased, considering the STs. As shown in **Table 1** and **Figure 2**, the pigmented and non-pigmented isolates were selected in pair from the same STs to ensure the equivalence between their phenotype and genotype. All the STs conferring both pigmented and non-pigmented isolates have been covered in the tested isolates. The genotypes of the 4 *S. argenteus* are different from those of the previous 61, thus, they were reclassified as Type 62 (3621, t12782), 63 (1850, t6103), 64 (2250, t5078) and 65 (2250, t7960).

Since there is a phylogenetic difference between *S. argenteus* and *S. aureus*, it is hypothesized that *S. argenteus* lacks the regulation systems to make a rational use of STX in response to those oxidation-based stress (Tong et al., 2013). Thus, it was regarded that the lack of STX might reduce the virulence of isolates to some extent. While our findings suggest that some non-pigmented *S. aureus* and *S. argenteus* strains did not show a reduced virulence in murine sepsis models, which is consistent with Tong et al. (2013). Although the reasons for the high virulence of non-pigmented isolates remain to be elucidated, our finding demonstrated that STX is not necessary for infection in the lineage of *S. aureus*.

There are lots of studies that aim to evaluate the different virulence between pigmented and non-pigmented strains. Some studies seem to support the hypothesis, for example, non-pigmented mutants had been used to compare with the corresponding wild-type (WT) strains, finding that it is more susceptible to desiccation and to linolenic acid (Wieland, 1994); compared with the WT bacterium, a *S. aureus* mutant with disrupted carotenoid biosynthesis is more susceptible to reactive oxygen species and neutrophil-based killing, and has mild pathogenicity in a mouse subcutaneous abscess model (Lan et al., 2010), however, other studies got the opposite results. For example, Tong et al. transferred Ptx-*crtOPQMN* to *S. argenteus*, which resulted in STX production and increased resistance to oxidative stress *in vitro*, however, neither resistance to neutrophil killing nor *in vivo* virulence was increased (Tong et al., 2013). Thus, it is unclear that if the virulence properties of non-pigmented *S. aureus* strains differ from those of pigmented due to its STX deficiency.

All in one, we came to the conclusion that there is no direct relationship between the presence of STX and genotype as well as the toxicity of the naturally isolated *S. aureus* strains. Non-pigmented isolates have the similar virulence with pigmented ones. Although blocking the biosynthesis of STX has been put forward as a novel potential anti-virulence strategy, the non-pigmented *S. aureus*, especially the *crtOPQMN* gene-deficient *S. argenteus* strains did not show a reduced virulence in the murine model. Thus it is reasonable to speculate that for better therapeutic effect against *S. aureus* infections, the *crtOPQMN* gene cluster involved in STX biosynthesis might need to be combined with multiple targets or treatments when being applied in alternative anti-virulence therapies.

## Author Contributions

JZ completed the quantification of pigment, MLST and *spa* typing, detection of virulence factors, and murine infection assay. YS collected and characterized the staphylococcal isolates, and analyzed their crtOPQMN genes. DZ helped to finish the MLST and *spa* typing, and murine infection assay. FJ helped to finish the quantification of pigment and detection of virulence factors. HZ helped to finish the quantification of pigment, and MLST and *spa* typing. CS designed the project, completed the data analysis, and prepared the manuscript.

## Conflict of Interest Statement

The authors declare that the research was conducted in the absence of any commercial or financial relationships that could be construed as a potential conflict of interest.

## Footnotes

1 http://saureus.mlst.net/

2 http://spaserver.ridom.de/
