# Supplementary material for: Genetic and Virulent Difference Between Pigmented and Non-pigmented Staphylococcus aureus
Source: Front Microbiol. 2018 Apr 3;9:598. doi: 10.3389/fmicb.2018.00598 (PMC5891619; doi:10.3389/fmicb.2018.00598)
Supplement: Supplementary file 1 [file Table_1.DOCX]

Supplementary Material

Genetic and Virulent Difference between Pigmented and Non-pigmented *Staphylococcus aureus*

Jing Zhang^1,†^, Yujuan Suo^2,†^, Daofeng Zhang^1^, Fangning Jin^1^, Hang Zhao^1^, Chunlei Shi^1,*^

^1^MOST-USDA Joint Research Center for Food Safety, School of Agriculture and Biology, Shanghai Jiao Tong University, Shanghai, P. R. China

^2^Institute of Agro-Food Standard and Testing Technology, Shanghai Academy of Agricultural Science, Shanghai, P. R. China

**^†^**These authors contribute equally to the study.

*** Correspondence:**

Prof. Chunlei Shi, Ph.D.

clshi@sjtu.edu.cn

**Table S1.** Primers used for PCR amplification of toxin genes in *S.aureus*.

| **Gene** | **Name** | **Sequence (5’---3’)** | **Size (bp)** | **Reference** |
| --- | --- | --- | --- | --- |
| *spa* | Spa-F | AATGCTGCGCAACACGATG | 1395 | This study |
|  | Spa-R | CGCTGCACCTAACGCTAATG |  |  |
| *sea* | Sea-F | ATTAACCGAAGGTTCTGTAGA | 552 | Xie et al., 2011 |
|  | Sea-R | TTGCGTAAAAAGTCTGAATT |  |  |
| *seb* | Seb-F | TGTATGTATGGAGGTGTAAC | 270 | Sharma et al., 2000 |
|  | Seb-R | ATAGTGACGAGTTAGGTA |  |  |
| *sec* | Sec-F | ACCAGACCCTATGCCAGATG | 371 | Cremonesi et al., 2005 |
|  | Sec-R | TCCCATTATCAAAGTGGTTTCC |  |  |
| *sed* | Sed-F | CTAGTTTGGTAATATCTCCT | 317 | Johnson et al., 1991 |
|  | Sed-R | TAATGCTATATCTTATAGGG |  |  |
| *see* | See-F | TAGATAAAGTTAAAACAAGC | 170 | Johnson et al., 1991 |
|  | See-R | TAACTTACCGTGGACCCTTC |  |  |
| *seg* | Seg-F | CCACCTGTTGAAGGAAGAGG | 432 | Cremonesi et al., 2005 |
|  | Seg-R | TGCAGAACCATCAAACTCGT |  |  |
| *seh* | Seh-F | CACATCATATGCGAAAGCAGA | 617 | Xie et al., 2011 |
|  | Seh-R | CCTTTTAAATCATAAATGTCGAATGA |  |  |
| *sei* | Sei-F | CTCAAGGTGATATTGGTGTAGG | 529 | Cremonesi et al., 2005 |
|  | Sei-R | CAGGCAGTCCATCTCCTGTA |  |  |
| *sej* | Sej-F | CAGCGATAGCAAAAATGAAACA | 426 | Rosec & Gigaud, 2002 |
|  | Sej-R | TCTAGCGGAACAACAGTTCTGA |  |  |
| *sek* | Sek-F | CGCTCAAGGCGATATAGGAA | 570 | Srinivasan et al., 2006 |
|  | Sek-R | GGTAACCCATCATCTCCTGTGT |  |  |
| *sel* | Sel-F | CACCAGAATCACACCGCTTA | 240 | Cremonesi et al., 2005 |
|  | Sel-R | CTGTTTGATGCTTGCCATTG |  |  |
| *sem* | Sem-F | CTATTAATCTTTGGGTTAATGGAGAAC | 300 | Jarraud et al., 2002 |
|  | Sem-R | TTCAGTTTCGACAGTTTTGTTGTCAT |  |  |
| *sen* | Sen-F | ATGAGATTGTTCTACATAGCTGCAAT | 680 | Jarraud et al., 2002 |
|  | Sen-R | AACTCTGCTCCCACTGAAC |  |  |
| *seo* | Seo-F | AGTTTGTGTAAGAAGTCAAGTGTAGA | 180 | Jarraud et al., 2002 |
|  | Seo-R | ATCTTTAAATTCAGCAGATATTCCATCTAAC |  |  |
| *sep* | Sep-F | GAATTGCAGGGAACTGCTTT | 537 | Srinivasan et al., 2006 |
|  | Sep-R | ACCAACCGAATCACCAGAAG |  |  |
| *seq* | Seq-F | GAACCTGAAAAGCTTCAAGGA | 509 | Srinivasan et al., 2006 |
|  | Seq-R | CCAGTTCCGGTGTAAAACAAA |  |  |
| *ser* | Ser-F | TTCAGTAAGTGCTAAACCAGATCC | 367 | Hwang et al., 2007 |
|  | Ser-R | CTGTGGAGTGCATTGTAACGCC |  |  |
| *ses* | Ses-F | TTCAGAAATAGCCAATCATTTCAA | 195 | Ono et al., 2008 |
|  | Ses-R | CCTTTTTGTTGAGAGCCGTC |  |  |
| *set* | Set-F | GGTGATTATGTAGATGCTTGGG | 170 | Ono et al., 2008 |
|  | Set-R | TCGGGTGTTACTTCTGTTTGC |  |  |
| *seu* | Seu-F | ATGGCTCTAAAATTGATGGTTCTA | 409 | Hwang et al., 2007 |
|  | Seu-R | GCCAGACTCATAAGGCGAACTA |  |  |
| *sey* | Sey-F | GGATCCAAAACAACTGGATTGATTA | 599 | Ono et al., 2015 |
|  | Sey-R | GTCGACCTATTTCATATAAATATCT |  |  |
| *tsst-1* | Tsst-F | TGCAAAAGCATCTACAAACGA | 499 | Srinivasan et al., 2006 |
|  | Tsst-R | TGTGGATCCGTCATTCATTG |  |  |
| *eta* | Eta-F | ACTGTAGGAGCTAGTGCATTTGT | 190 | Jarraud et al., 2002 |
|  | Eta-R | TGGATACTTTTGTCTATCTTTTTCATCAAC |  |  |
| *etb* | Etb-F | CAGATAAAGAGCTTTATACACACATTAC | 612 | Jarraud et al., 2002 |
|  | Etb-R | AGTGAACTTATCTTTCTATTGAAAAACACTC |  |  |
| *etd* | Etd-F | CGCAAATACATATGAAGAATCTGA | 452 | Nakaminami et al., 2008 |
|  | Etd-R | TGTCACCTTGTTGCAAATCTATAG |  |  |
| *PVL* | PVL-1 | ATCATTAGGTAAAATGTCTGGACATGATCCA | 433 | Jarraud et al., 2002 |
|  | NPVL-2 | GCATCAASTGTATTGGATAGCAAAAGC |  |  |
| *vWbp* | vWbp-F | ATTGCTAGTTTTATCATTGGG | 1180 | This study |
|  | vWbp-R | ATGACTTTCACCATTTAATCC |  |  |
| *sak* | Sak-F | CATCAAGTTCATTCGACAAA | 355 | This study |
|  | Sak-R | CTGATAAATCTGGGACAACAA |  |  |
| *cna* | Cna-F | AACATCTGGGAATAAATCAACG | 713 | This study |
|  | Cna-F | TATAGTCTCCGCTAGGCAACG |  |  |
| *chp* | Chp-F | TTAGCAACAACAGTTTTAGCA | 320 | This study |
|  | Chp-R | TTAGCAACAACAGTTTTAGCA |  |  |
| *crt4* | Crt4-F | TATCGCCAAATGTCTGTARGA | - | Zhang et al., 2016 |
|  | Crt4-R | GTGGGACCCATATCAAATGTA |  |  |
| *crt5* | Crt5-F | GGCAGCCCGTATTGCTTCTCA | - | Zhang et al., 2016 |
|  | Crt5-R | AAACCGAATGCCGAACCAAAT |  |  |
| *crt6* | Crt6-F | CGGTATAGATATTGATGTGAC | - | Zhang et al., 2016 |
|  | Crt6-R | AAATCTACAATRGCTTGTTTG |  |  |

**References**

Cremonesi, P., Luzzana, M., Brasca, M., Morandi, S., Lodi, R., Vimercati, C., et al. (2005). Development of a multiplex PCR assay for the identification of Staphylococcus aureus enterotoxigenic strains isolated from milk and dairy products. *Molecular and Cellular Probes, 19*(5), 299-305. doi: 10.1016/j.mcp.2005.03.002

Hwang, S. Y., Kim, S. H., Jang, E. J., Kwon, N. H., Park, Y. K., Koo, H. C., et al. (2007). Novel multiplex PCR for the detection of the Staphylococcus aureus superantigen and its application to raw meat isolates in Korea. *International Journal of Food Microbiology, 117*(1), 99-105. doi: 10.1016/j.ijfoodmicro.2007.02.013

Jarraud, S., Mougel, C., Thioulouse, J., Lina, G., Meugnier, H., Forey, F., et al. (2002). Relationships between Staphylococcus aureus genetic background, virulence factors, agr groups (alleles), and human disease. *Infection and Immunity, 70*(2), 631-641.

Johnson, W. M., Tyler, S. D., Ewan, E. P., Ashton, F. E., Pollard, D. R., & Rozee, K. R. (1991). Detection of genes for enterotoxins, exfoliative toxins, and toxic shock syndrome toxin 1 in Staphylococcus aureus by the polymerase chain reaction. *Journal of Clinical Microbiology, 29*(3), 426-430.

Nakaminami, H., Noguchi, N., Ikeda, M., Hasui, M., Sato, M., Yamamoto, S., et al. (2008). Molecular epidemiology and antimicrobial susceptibilities of 273 exfoliative toxin-encoding-gene-positive Staphylococcus aureus isolates from patients with impetigo in Japan. *Journal of Medical Microbiology, 57*(Pt 10), 1251-1258. doi: 10.1099/jmm.0.2008/002824-0

Ono, H. K., Omoe, K., Imanishi, K., Iwakabe, Y., Hu, D. L., Kato, H., et al. (2008). Identification and characterization of two novel staphylococcal enterotoxins, types S and T. *Infection and Immunity, 76*(11), 4999-5005. doi: 10.1128/IAI.00045-08

Ono, H. K., Sato'o, Y., Narita, K., Naito, I., Hirose, S., Hisatsune, J., et al. (2015). Identification and Characterization of a Novel Staphylococcal Emetic Toxin. *Appllied and Environmental Microbiology, 81*(20), 7034-7040. doi: 10.1128/AEM.01873-15

Rosec, J. P., & Gigaud, O. (2002). Staphylococcal enterotoxin genes of classical and new types detected by PCR in France. *International Journal of Food Microbiology, 77*(1-2), 61-70.

Sharma, N. K., Rees, C. E., & Dodd, C. E. (2000). Development of a single-reaction multiplex PCR toxin typing assay for Staphylococcus aureus strains. *Appllied and Environmental Microbiology, 66*(4), 1347-1353.

Srinivasan, V., Sawant, A. A., Gillespie, B. E., Headrick, S. J., Ceasaris, L., & Oliver, S. P. (2006). Prevalence of enterotoxin and toxic shock syndrome toxin genes in Staphylococcus aureus isolated from milk of cows with mastitis. *Foodborne Pathog Dis, 3*(3), 274-283. doi: 10.1089/fpd.2006.3.274

Xie, Y., He, Y., Gehring, A., Hu, Y., Li, Q., Tu, S. I., et al. (2011). Genotypes and toxin gene profiles of Staphylococcus aureus clinical isolates from China. *PLoS One, 6*(12), e28276. doi: 10.1371/journal.pone.0028276

Zhang, D. F., Xu, X., Song, Q., Bai, Y., Zhang, Y., Song, M., et al. (2016). Identification of Staphylococcus argenteus in Eastern China based on a nonribosomal peptide synthetase (NRPS) gene. *Future Microbiol, 11*, 1113-1121. doi: 10.2217/fmb-2016-0017
